# Topological phonons in oxide perovskites controlled by light

**DOI:** 10.1126/sciadv.abd1618

**Published:** 2020-11-11

**Authors:** Bo Peng, Yuchen Hu, Shuichi Murakami, Tiantian Zhang, Bartomeu Monserrat

**Affiliations:** 1 Cavendish Laboratory, University of Cambridge, JJ Thomson Avenue, Cambridge CB3 0HE, UK.; 2 Department of Chemistry, University of Cambridge, Lensfield Road, Cambridge CB2 1EW, UK.; 3 Department of Physics, Tokyo Institute of Technology, Ookayama, Meguro-ku, Tokyo 152-8551, Japan.; 4 Tokodai Institute for Element Strategy, Tokyo Institute of Technology, Nagatsuta, Midori-ku, Yokohama, Kanagawa 226-8503, Japan.; 5 Department of Materials Science and Metallurgy, University of Cambridge, 27 Charles Babbage Road, Cambridge CB3 0FS, UK.

## Abstract

Perovskite oxides exhibit a rich variety of structural phases hosting different physical phenomena that generate multiple technological applications. We find that topological phonons—nodal rings, nodal lines, and Weyl points—are ubiquitous in oxide perovskites in terms of structures (tetragonal, orthorhombic, and rhombohedral), compounds (BaTiO_3_, PbTiO_3_, and SrTiO_3_), and external conditions (photoexcitation, strain, and temperature). In particular, in the tetragonal phase of these compounds, all types of topological phonons can simultaneously emerge when stabilized by photoexcitation, whereas the tetragonal phase stabilized by thermal fluctuations only hosts a more limited set of topological phonon states. In addition, we find that the photoexcited carrier concentration can be used to tune the topological phonon states and induce topological transitions even without associated structural phase changes. Overall, we propose oxide perovskites as a versatile platform in which to study topological phonons and their manipulation with light.

## Teaser

Perovskites have it all: light-driven topological phonons added to the long list of properties of this family of materials.

## INTRODUCTION

In the family of topological materials, semimetals have an important place in the study of topological order because they exhibit topologically protected surface states (*1*), an anomalous bulk transport phenomenon known as the “quantum anomaly” (*2*, *3*), diversified classifications like nodal rings and lines (*4*–*6*), and Weyl and Dirac points (*7*–*11*), and they serve as a platform for obtaining various other topological states, such as topological (crystalline) insulators (*12*) and Chern insulators (*13*). Beyond electronic structures, topological degeneracies in the spectra of other quasiparticles such as excitons, magnons, and phonons have drawn wide attention in the past decade (*14*–*18*). One advantage of the latter group is that excitation of these quasiparticles depends only on the energy of the external probe, rather than being restricted to the bands near the Fermi level like in the case of electrons. Among these quasiparticles, phonons are of particular interest as basic emergent bosonic excitations associated with lattice vibrations (*19*–*24*) and contribute to many physical processes, such as conventional superconductivity (*25*, *26*) and the thermal Hall effect (*27*–*30*). Thus, studies of topological phonons, and especially on the control of topological phonons, are becoming an important field in condensed matter physics.

Oxide perovskites have gained popularity as a material system in the past decades due to hosting multiple competing phases including ferroelectric (*31*), magnetic (*32*), or superconducting (*26*),as well as exotic excitations such as skyrmions (*33*, *34*). In addition, these competing phases are particularly sensitive to external stimuli, including temperature (*35*), strain (*36*), pressure (*37*), and composition (*38*). More recently, theoretical and experimental evidence has demonstrated that light is yet another means by which it is possible to control the crystal symmetry of perovskites (*39*–*43*). As topological order is intimately related to symmetry, the versatility of the perovskite family makes these structures promising platforms to also explore topological properties. However, most oxide perovskites are large gap insulators (for example, BaTiO_3_, SrTiO_3_, and PbTiO_3_), and as a consequence, they exhibit no electronic topological states.

In this work, we show that the phonon spectrum of multiple noncentrosymmetric perovskites can host three types of topological states—topological nodal rings, nodal lines, and Weyl points—suggesting that topological phonons are pervasive in different structural phases of oxide perovskites. Using the tetragonal BaTiO_3_ phase as a prototype, we show that all these types of topological phonon emerge simultaneously when this phase is stabilized by photoexcitation. In addition, topological order can be controlled with the photoexcited carrier density, driving topological transitions without any associated structural phase change, including the creation and annihilation of Weyl phonons and switching between nodal-ring and nodal-line phonons. In contrast, when the tetragonal phase of BaTiO_3_ is stabilized by thermal fluctuations, it only exhibits a more limited number of topological states. This is because the long-range Coulomb interaction leads to a large energy splitting between the longitudinal and transverse optical phonons (LO-TO splitting), reducing the possibility of topological degeneracies between these bands. The photoexcitation route eliminates this problem because photoexcited carriers can screen long-range interactions.

## RESULTS AND DISCUSSION

### Crystal structures and structural phase transitions in BaTiO_3_

**Fig. 1:**
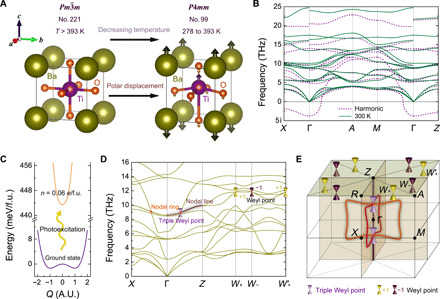
Crystal structure and phonon dispersion of tetragonal BaTiO_3_. (**A**) Crystal structures of the high-temperature cubic phase (no. 221, $\mathrm{Pm}\bar{3}m$) and of the room-temperature tetragonal phase (no. 99, *P4mm*) of BaTiO_3_. (**B**) Phonon dispersion of tetragonal BaTiO_3_ at the harmonic level and at 300 K including anharmonic vibrations. (**C**) Ground-state and excited-state potential energy surfaces along the Γ point phonon mode that is imaginary within the harmonic approximation. A.U., arbitrary unit. (**D**) Phonon dispersion of tetragonal BaTiO_3_ at *n* = 0.06 *e*/f.u. (**E**) Bulk Brillouin zone at *n* = 0.06 *e*/f.u. with two nodal rings on the *q_x_* = 0 and *q_y_* = 0 mirror planes (orange circles, formed by the 10th and 11th bands), one nodal line along the Γ-*Z* high-symmetry line (brown line, formed by the 10th and 11th bands), one pair of triple Weyl points at the intersection points between the nodal rings and nodal line (violet cones, formed by the 10th, 11th, and 12th bands), and four pairs of Weyl points on the *q_z_* = π plane (purple and yellow cones, formed by the 10th and 11th bands).

As one of the most studied perovskite materials, BaTiO_3_ has a cubic ABO_3_-type crystal structure at temperatures above 393 K, with the A cation (Ba^2+^) at the corners and the B cation (Ti^4+^) at the center of an octahedral cage of oxygen atoms. Upon cooling, BaTiO_3_ undergoes an inversion symmetry breaking, giving rise to a tetragonal phase with a deformation along the [001] direction (*44*). As shown in Fig. 1A, the phase transition breaks inversion symmetry by the polar displacements of Ti and O atoms along the *z* direction, which changes the space group from $\mathrm{Pm}\bar{3}m$ (no. 221) to *P*4*mm* (no. 99). With further cooling, an orthorhombic *Amm*2 phase appears below 278 K and a rhombohedral *R*3*m* phase follows below 183 K. As a result of these temperature-driven phase transitions, the room temperature tetragonal phase exhibits imaginary harmonic phonon modes, as shown in Fig. 1B. The inclusion of anharmonic vibrations (*45*) stabilizes the tetragonal structure at 300 K, also shown in Fig. 1B.

In addition to temperature, multiple strategies have traditionally been proposed to control structural phase transitions in perovskites, including strain (*36*), pressure (*32*, *37*), and composition (*38*). More recently, it has been shown that photoexcitation provides an alternative route to stabilizing multiple perovskite phases (*39*–*43*). Photoexcitation is relatively economical and can be easier to control compared to other strategies such as heating to change temperature, material synthesis to change composition or strain, or external pressure. Inspired by these recent discoveries, we find that photoexcitation can also stabilize the imaginary phonon modes of the tetragonal phase of BaTiO_3_: Fig. 1C shows that the double-well potential energy curve along the imaginary phonon mode of amplitude *Q* at the Γ point becomes a single well upon illumination, indicating the stabilization of the crystal structure. The underlying physics is that changing the carrier concentration *n* induced by photoexcitation leads to changes in the potential energy experienced by the ions, which, in turn, changes the interatomic force constants and the phonon dispersion. For 0.04 < *n* < 0.10 *e*/f.u. (electron per formula unit), the *P*4*mm* phase is not only dynamically stable but also thermodynamically more stable than the cubic $\mathrm{Pm}\bar{3}m$ phase; whereas for *n* > 0.10 *e*/f.u., the *P*4*mm* phase relaxes to the cubic phase as the latter becomes thermodynamically more stable (*39*). The relative energy between the two phases and their corresponding lattice constants under illumination are shown in the Supplementary Materials. Figure 1D shows the phonon dispersion with a photoexcited carrier density of *n* = 0.06 *e*/f.u., confirming the dynamical stabilization of the tetragonal phase.

### Topological phonons in BaTiO_3_

**Fig. 2:**
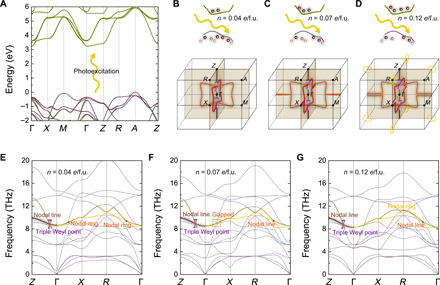
Nodal lines and nodal rings in tetragonal BaTiO_3_ controlled by light. (**A**) Schematic of the photoexcitation process in tetragonal BaTiO_3_. (**B** to **D**) Nodal lines and nodal rings formed by the 10th and 11th phonon branches in the bulk Brillouin zone for photoexcited carrier concentrations of 0.04, 0.07, and 0.12 *e*/f.u. The corresponding phonon dispersions are shown in (**E** to **G**).

As a large gap insulator, BaTiO_3_ displays trivial topology in its electronic band structure (Fig. 2A). However, there are three types of topological states in its phonon dispersion, which are nodal rings, nodal lines, and Weyl points. Since the unit cell of tetragonal BaTiO_3_ has five atoms, there are 15 branches in the phonon spectrum. Band inversion between the 10th and 11th bands (labeled by increasing energy) along the *X*-Γ high-symmetry line is protected by *M_x_* symmetry, which restricts the band crossing to a one-dimensional continuous ring/line on the mirror-invariant plane (*46*). Therefore, a nodal ring is formed on the *q_x_* = 0 plane. Because of the existence of an additional *C*_4*z*_ symmetry, there is another nodal ring located on the *q_y_* = 0 plane, related to the one on the *q_x_* = 0 plane by *C*_4*z*_ symmetry (orange circles in Fig. 1E). The combination of *C*_4*z*_ and mirror symmetries also brings out another type of topological phonon in BaTiO_3_: an endless nodal line along the Γ-*Z* direction (brown line in Fig. 1E). Intersection points between these two nodal rings and the nodal line form two triple Weyl points located along the *Z*′(0,0,−π)-Γ-*Z*(0,0,π) line. Another topological feature of the band crossings between the 10th and 11th bands are eight Weyl points on the *q_z_* = π plane (purple and yellow cones in Fig. 1E). These eight Weyl points, located at generic momenta on the *q_z_* = π plane, are not protected by any crystalline symmetry because of the breaking of the *M_z_* symmetry in the tetragonal lattice, so we need to apply more advanced symmetry-based indicator theory to diagnose the topology.

**Fig. 3:**
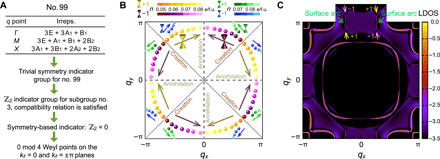
Weyl points in tetragonal BaTiO_3_ controlled by light. (**A**) Diagnosis process for the eight Weyl points in BaTiO_**3**_ using symmetry-based indicator theory for the lowest 10 bands. (**B**) Evolution of the Weyl points on the *q_z_* = π plane with increasing *n* from 0.050 to 0.085 *e*/f.u. (**C**) Phonon surface arcs for a phonon frequency of 11.42 THz at *n* = 0.07 *e*/f.u. LDOS, local density of states.

To gain a better understanding of the topological nature of the eight Weyl points present in photoexcited tetragonal BaTiO_3_, we explore the existence of Weyl points from the perspective of topology, as shown in Fig. 3A, diagnosing topological degeneracies by using symmetry-based indicators from subgroups (*47*–*49*). After obtaining the symmetry data at Γ, *M*, and *X*, we note that the symmetry-based indicator group for space group no. 99 is a trivial one and that the symmetry data at high-symmetry momenta does not satisfy the compatibility condition. Thus, we need to find a subgroup that has a nontrivial symmetry-based indicator group and satisfies the compatibility condition (*23*, *50*). Space group no. 99 has five subgroups with the same size of unit cell, namely, no. 75 (*P*4), no. 25 (*Pmm*2), no. 6 (*Pm*), no. 3 (*P*2), and no. 1 (*P*1). Among them, no. 3 is the maximal subgroup that has a nontrivial symmetry-based indicator group ℤ_2_ and satisfies the compatibility condition. Thus, subgroup no. 3 is used for further diagnosis. Space group no. 3 only has a generator of *C*_2*z*_ rotation symmetry, so the symmetry-based indicator group ℤ_2_ corresponds to the *z*_2_ Berry phase of the loop enclosing half of the *q_z_* = 0 [*X*(π,0,0)-*M*(π,π,0)*-M*′(−π,π,0)*-X*′(−π,0,0)*-X*(π,0,0)] or *q_z_* = π [*R*(π,0,π)*-A*(π,π,π)*-A*′(−π,π,π)*-R*′(−π,0,π)*-R*(π,0,π)] plane. If *z*_2_ = 0, then there will be 0 mod 4 Weyl points on the *q_z_* = 0 and *q_z_* = π planes. Our calculations deliver eight Weyl points in total, corresponding to the case ℤ_2_ = 0. Furthermore, considering the additional mirror symmetries in space group no. 99, the eight Weyl points on the *q_z_* = π plane are related by *M_x_* and *M_y_* symmetries: four of them with left-hand chirality while the other four with right-hand chirality.

### Controlling topological phonons by light

As discussed above, both photoexcitation with 0.04 < *n* < 0.10 *e*/f.u. and temperatures between 278 and 393 K can stabilize the tetragonal phase of BaTiO_3_, as shown in Fig. 1 (B and D). However, a main difference is that without photoexcitation, the long-range Coulomb interaction leads to a large LO-TO splitting (*51*), which tends to lift band degeneracies, as shown in Fig. 1B. Consequently, the eight conventional Weyl points and the two triple Weyl points that we find in illuminated BaTiO_3_ are not observed in temperature-stabilized tetragonal BaTiO_3_ at 300 K (although the presence of nodal-line and nodal-ring phonons is confirmed in the Supplementary Materials). In contrast, under illumination, the photoexcited carriers lead to a strong free-carrier screening that suppresses the LO-TO energy splitting and facilitates the appearance of Weyl phonons in tetragonal BaTiO_3_. Another advantage of the photoexcitation route is that, as discussed next, it can drive the phononic system between different topological quantum states, including switching between nodal-ring and nodal-line phonons, controlling the position of Weyl points in momentum space, and creating and annihilating these topological states.

As discussed above, changing the photoexcited carrier density brings about not only the transition between nodal rings and nodal lines but also the creation of new nodal rings. As shown in Fig. 2 (B and C), increasing the photoexcited carrier density from *n* = 0.04 *e*/f.u. to *n* = 0.07 *e*/f.u. drives the two nodal rings to become two nodal lines, and the corresponding phonon spectra are shown in Fig. 2 (E and F). Further increasing *n* to 0.12 *e*/f.u. (under which conditions the tetragonal structure becomes cubic) leads to the formation of two nodal rings around the *R* point on the *q_x_* = 0 and *q_y_* = 0 planes, as shown in Fig. 2 (D and G). Thus, by changing *n*, nodal rings in the phonon spectra can be created or transformed into nodal lines.

In addition to nodal lines and nodal rings, Weyl phonons are also sensitive to the photoexcited carrier concentration *n*. Figure 3B shows the evolution of eight pairs of Weyl points on the *q_z_* = π plane when increasing *n* from 0.050 to 0.085 *e*/f.u. (four extra pairs of Weyl points emerge when 0.0695 < *n* < 0.0825 *e*/f.u.). Below 0.050 *e*/f.u., BaTiO_3_ exhibits no Weyl points between the 10th and 11th branches in its phonon spectrum. At about *n* = 0.050 *e*/f.u., four pairs of Weyl points are created around the *q_x_* = *q_y_* and *q_x_* = −*q_y_* planes (orange and maroon dots in Fig. 3B). As *n* increases, the Weyl points in each pair with different chirality move away from each other and head to the *q_x_* = 0 and *q_y_* = 0 planes (yellow and purple arrows in Fig. 3B). Once *n* reaches 0.0815 *e*/f.u., Weyl points meet on the *q_x_* = 0 and *q_y_* = 0 planes and annihilate in pairs (light yellow and light magenta dots in Fig. 3B). In addition, another four pairs of Weyl points of opposite chirality are created around the *q_x_* = *q_y_* and *q_x_* = −*q_y_* planes at *n* = 0.0695 *e*/f.u. and annihilate in pairs on the *q_x_* = π and *q_y_* = π planes when *n* reaches 0.0825 *e*/f.u. (blue and green dots in Fig. 3B). Since no band inversion happens at any high-symmetry points in this process, the Berry phase of the loop *R*-*A*-*A*′-*R*′-*R* on the *q_z_* = π plane remains zero, which is consistent with 0 mod 4 Weyl points on the *q_z_* = π plane with ℤ_2_ = 0 (Fig. 3A).

To have a better understanding of the Weyl phonons in tetragonal BaTiO_3_, we calculate the surface local density of states (LDOS) from the imaginary part of the surface Green’s function (*52*). The surface LDOS in Fig. 3C is calculated along the (001) direction at 11.42 THz and *n* = 0.07 *e*/f.u. The four pairs of Weyl points connect via surface arcs crossing the Brillouin zone boundaries so that each surface arc starts from one Weyl point with a positive monopole charge and ends at another with a negative one. Overall, by modulating *n*, we can control the creation and annihilation of Weyl points and the length of surface arcs in tetragonal BaTiO_3_.

### Topological phonons in PbTiO_3_

**Fig. 4:**
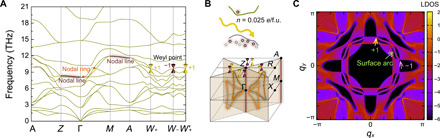
Topological phonons in tetragonal PbTiO_3_. (**A**) Phonon dispersion of tetragonal *P*4*mm* PbTiO_**3**_ at *n* = 0.025 *e*/f.u. (**B**) Bulk Brillouin zone at *n* = 0.025 *e*/f.u. with four nodal rings on the *q_x_* = *q_y_* and *q_x_* = −*q_y_* planes (orange circles), two nodal lines along the Γ-*Z* and *M*-*A* high-symmetry lines (brown line), and eight Weyl points located on the *q_z_* = π plane (purple and yellow cones). All these three types of topological phonons are composed of the 10th and 11th bands. (**C**) Phonon surface arcs for a phonon frequency of 9.63 THz at *n* = 0.025 *e*/f.u.

Multiple types of topological phonons can also be found in other perovskites with the same *P*4*mm* space group, and here, we study PbTiO_3_ and SrTiO_3_ as additional examples. PbTiO_3_ has a tetragonal *P*4*mm* structure for *T* < 600 K and a cubic structure above that temperature. Although the *P*4*mm* phase has the lowest energy in a wide photoexcited density range *n* < 0.125 *e*/f.u. (*39*), it becomes dynamically unstable when *n* > 0.05 *e*/f.u. (shown in the Supplementary Materials). We calculate the excited-state phonon dispersion at *n* = 0.025 *e*/f.u., as shown in Fig. 4A. Similar to BaTiO_3_, PbTiO_3_ also has nodal rings, nodal lines, and Weyl points between the 10th and 11th bands, but the triple Weyl points no longer exist, as the nodal rings and the nodal line do not touch. The unique band crossings along the Γ-*M* high-symmetry line bring about four nodal rings on the *q_x_* = *q_y_* and *q_x_* = −*q_y_* planes protected by *M_xy_* and $M_{x\bar{y}}$ symmetries. The endless nodal lines along the Γ-*Z* and *M*-*A* directions are robust as well, as they are protected by the *C*_4*z*_ symmetry.

The surface LDOS in Fig. 4C is calculated along the (001) direction at 9.63 THz with *n* = 0.025 *e*/f.u. Four pairs of Weyl points, created at *n* = 0.005 *e*/f.u., connect with each other via surface arcs crossing the *q_x_* = *q_y_* and *q_x_* = −*q_y_* planes. We note that the location of these four pairs of Weyl points can also be modulated by changing the photoexcitation density until the lattice becomes dynamically unstable for *n* > 0.05 *e*/f.u.

### Topological phonons in SrTiO_3_

**Fig. 5:**
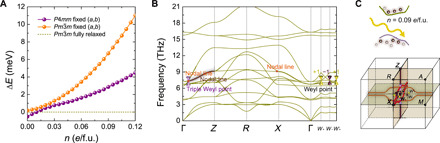
Topological phonons in tetragonal SrTiO_3_. (**A**) Relative energy difference between the *P*4*mm* and $\mathrm{Pm}\bar{3}m$ phases with fixed in-plane lattice constants *a* and *b*, with the energy of the fully relaxed $\mathrm{Pm}\bar{3}m$ phase set to zero. (**B**) Phonon dispersion of tetragonal *P*4*mm* SrTiO_3_ at *n* = 0.09 *e*/f.u. (**C**) Bulk Brillouin zone at *n* = 0.09 *e*/f.u. with four nodal lines in total on the *q_x_* = 0 and *q_y_* = 0 planes (orange lines), one nodal line along the Γ-*Z* high-symmetry line (brown line), one pair of triple Weyl points at the intersection points between the three nodal lines (violet cones), and four pairs of Weyl points on the *q*_***z***_ = 0 plane (purple and yellow dots).

SrTiO_3_ has a different phase diagram compared to BaTiO_3_ and PbTiO_3_. At low temperatures, it exhibits a centrosymmetric tetragonal phase of space group *I*4/*mcm* and undergoes a phase transition to a cubic phase above 106 K. The *P*4*mm* phase can only be accessed with small negative strains (*53*, *54*). The in-plane lattice constants *a* and *b* of *P*4*mm* SrTiO_3_ increase upon photoexcitation. Therefore, by fixing *a* and *b* to the ones in the dark, we can induce in-plane negative strain in illuminated SrTiO_3_. This can be realized experimentally by growing SrTiO_3_ on an appropriate substrate. As shown in Fig. 5A, after fixing the in-plane lattice constants *a* and *b* and relaxing only the out-of-plane lattice constant *c* in the *P*4*mm* phase, we find that the tetragonal phase can have a lower energy than the cubic $\mathrm{Pm}\bar{3}m$ phase.

*P*4*mm* SrTiO_3_ exhibits imaginary phonon modes in the dark, while adding a photoexcited charge density of *n* = 0.09 *e*/f.u. stabilizes the structure, as shown in Fig. 5B. Similar to BaTiO_3_, the *P*4*mm* phase of SrTiO_3_ has four nodal lines in total on the *q_x_* = 0 and *q_y_* = 0 mirror planes and one nodal line along the Γ-*Z* high-symmetry line in the bulk Brillouin zone. The intersection points of the three nodal lines form one pair of triple Weyl points. Different from BaTiO_3_, the four pairs of Weyl points are located on the *q_z_* = 0 plane rather than the *q_z_* = π plane. Weyl phonons in SrTiO_3_ can also be manipulated by photoexcitation, and the critical *n* for the annihilation of the Weyl points on the *q_x_* = 0 and *q_y_* = 0 planes is 0.105 *e*/f.u., which is similar to that in BaTiO_3_.

### Ubiquity of topological phonons in perovskite oxides

The existence and coexistence of different types of topological phonons, together with their tunability by light, occur in a variety of other structural phases of perovskites beyond the tetragonal phases investigated above. Topological phonons in the orthorhombic *Amm*2 and the rhombohedral *R*3*m* phases of BaTiO_3_ are shown in the Supplementary Materials. Furthermore, topological phonons in oxide perovskites can also be manipulated by other tuning parameters beyond photoexcitation, such as strain and temperature (for details, see the Supplementary Materials). Putting these insights together suggests that topological phonons are ubiquitous in the family of perovskite oxides, which, given their widespread use and versatility, provide a promising platform for exploring topological phonon physics and its interplay with other phases.

Given the proposed ubiquity of topological phonons in perovskites, it should be possible to directly identify them experimentally by looking for “isolated” band crossings, and the prototype materials detailed above are promising starting candidates. Topological bulk phonons can be measured by inelastic neutron scattering (*23*, *55*, *56*) and inelastic x-ray scattering (*20*), while the accompanying topological surface states can be detected by high-resolution electron energy loss spectroscopy (*57*). For light-induced topological phonons, although it may be difficult to illuminate the sample to maintain a constant photoexcited carrier density, transient photoexcitation could be used to observe the evolution of nodal rings, nodal lines, and Weyl points as a function of the time-dependent photoexcited carrier density.

In terms of phenomena, topological phonons with nonzero Berry curvature may contribute to the recently observed phonon thermal Hall effect in SrTiO_3_ (*30*, *58*). In addition, previously unknown phonon-phonon and electron-phonon scattering mechanisms could arise from topological phonons, which may provide new insights into the enhanced superconductivity of SrTiO_3_ (*26*, *59*). The emergence of Weyl phonons may be accompanied by other unique physical properties such as a pseudogauge field with a one-way propagating bulk mode (*60*–*62*), topological negative refraction (*18*), and nonlinear acoustic/optical responses (*63*, *64*), which can offer new routes for designing novel technologies like light-controlled neuromorphic computing in phononic systems (*65*, *66*). Our work shows that oxide perovskites provide a promising platform to explore all of these phenomena and applications.

We find that topological phonons are ubiquitous in oxide perovskites and that photoexcitation provides a promising route for their manipulation. As examples, the noncentrosymmetric tetragonal phases of three oxide perovskites (BaTiO_3_, PbTiO_3_, and SrTiO_3_) exhibit topological nodal rings, nodal lines, and Weyl points in their phonon spectra. We find that photoexcitation is the only way to obtain Weyl phonons in tetragonal BaTiO_3_ since the thermally stabilized tetragonal phase has a large LO-TO energy splitting in the phonon spectrum that prevents band crossings. By contrast, photoexcited carriers screen long-range interactions, suppressing the LO-TO energy splitting and facilitating the band crossings. We also find that the photoexcited carrier density can be used to tune the creation and annihilation of Weyl points and nodal rings/lines without any associated structural phase transitions. Topological phonons in oxide perovskites provide a promising platform to study physical phenomena, ranging from the phonon Hall effect to superconductivity, and may also offer new technological opportunities such as the realization of controllable topological quantum states for neuromorphic computing.

## METHODS

Density functional theory (DFT) calculations are performed using the Vienna Ab Initio Simulation Package (VASP) with the projector-augmented wave potential method (*67*, *68*). We use the generalized gradient approximation with the Perdew-Burke-Ernzerhof parameterization revised for solids as the exchange-correlation functional (*69*). A plane-wave basis set is used with a kinetic energy cutoff of 800 eV and a 7 × 7 × 7 *k*-mesh during structural relaxation, which is stopped when forces are below 10^−3^ eV/Å. The band structure of BaTiO_3_ is calculated using the HSE06 hybrid functional in the presence of spin-orbit coupling (*70*). Photoexcited carriers are simulated by promoting electrons from high-energy valence band states to low-energy conduction band states. This Δ self-consistent field (ΔSCF) method introduces noninteracting electron-hole pairs by changing the occupation numbers of the Kohn-Sham orbitals (*71*–*74*) and is computationally less demanding compared to other approaches like constrained DFT (*75*) and excited-state force calculations (*76*). Nevertheless, it gives consistent phonon spectra compared with those obtained with constrained DFT (*39*), as shown in the Supplementary Materials. The occupancies are fixed with a smearing of 0.01 eV.

The crystal structures in the dark and under photoexcitation are fully relaxed before the phonon calculations. We calculate the force constants with density functional perturbation theory (DFPT) (*77*) in a 2 × 2 × 2 supercell with a 5 × 5 × 5 *k*-mesh using VASP. The phonon dispersion is then obtained using Phonopy (*78*). We perform convergence tests on the supercell size between 2 × 2 × 2, 3 × 3 × 3, and 4 × 4 × 2, all confirming the existence of Weyl points in illuminated BaTiO_3_.

We calculate the chirality of Weyl points by using the Wilson loop method to calculate the Wannier charge center flow, i.e., the monopole charge of a Weyl point (*79*, *80*). The phonon surface states are obtained using surface Green’s functions as implemented in WannierTools (*52*). The finite-temperature phonon frequencies including anharmonic contributions are calculated using a self-consistent ab initio lattice dynamical method (*45*, *81*, *82*) in a 2 × 2 × 2 supercell. The self-consistent cycle is terminated after 240 iterations, when the difference in free energy is less than 0.2 meV and the space group symmetry is enforced on the resulting force constants. The Born effective charges are calculated using DFPT to obtain the LO-TO splitting in the phonon dispersion in the dark (*83*). Under illumination, the photoexcited electrons screen long-range interactions and no LO-TO splitting occurs.

## Supplementary Material

http://advances.sciencemag.org/cgi/content/full/6/46/eabd1618/DC1

Adobe PDF - abd1618_SM.pdf

Topological phonons in oxide perovskites controlled by light

## SUPPLEMENTARY MATERIALS

Supplementary material for this article is available at http://advances.sciencemag.org/cgi/content/full/6/46/eabd1618/DC1
