# Supplementary material for: Topological phonons in oxide perovskites controlled by light
Source: Sci Adv. 2020 Nov 11;6(46):eabd1618. doi: 10.1126/sciadv.abd1618 (PMC7673742; doi:10.1126/sciadv.abd1618)
Supplement: http://advances.sciencemag.org/cgi/content/full/6/46/eabd1618/DC1 [file supp_6_46_eabd1618__index.html]

Science Advances | Science AdvancesAAASSearchScience AdvancesMenu

## Supplementary Materials

# Topological phonons in oxide perovskites controlled by light

Bo Peng, Yuchen Hu, Shuichi Murakami, Tiantian Zhang, Bartomeu Monserrat

Download Supplement

**This PDF file includes:**

- Supplementary Text
- Figs. S1 to S9

**Files in this Data Supplement:**

- Adobe PDF - abd1618\_SM.pdf
