# Supplementary material for: Topological phonons in oxide perovskites controlled by light
Source: Sci Adv. 2020 Nov 11;6(46):eabd1618. doi: 10.1126/sciadv.abd1618 (PMC7673742; doi:10.1126/sciadv.abd1618)
Supplement: http://advances.sciencemag.org/cgi/content/full/6/46/eabd1618/DC1 [file supp_6_46_eabd1618__1.pdf]

Science Advances
AAAS

advances.sciencemag.org/cgi/content/full/6/46/eabd1618/DC1

Supplementary Materials for

Topological phonons in oxide perovskites controlled by light

Bo Peng, Yuchen Hu, Shuichi Murakami, Tiantian Zhang*, Bartomeu Monserrat*

*Corresponding author. Email: ttzhang@stat.phys.titech.ac.jp (T.Z.); bm418@cam.ac.uk (B.M.)

Published 11 November 2020, *Sci. Adv.* **6**, eabd1618 (2020)
DOI: 10.1126/sciadv.abd1618

This PDF file includes:

Supplementary Text
Figs. S1 to S9

Relative stability of $P4mm$ and $Pm\bar{3}m$ $BaTiO_3$ under illumination

As discussed in Ref. (*39*), the thermodynamically stable structures of $BaTiO_3$ under photoexcitation are the $R3m$ phase for a photoexcited carrier density of $n < 0.035$ e/f.u., the $P4mm$ phase for a photoexcited carrier density between $0.04 - 0.1$ e/f.u., and the cubic $Pm\bar{3}m$ phase for $n > 0.1$ e/f.u. Between $0.035 - 0.040$ e/f.u. all three $R3m$, $Amm2$, and $P4mm$ phases have very similar energies. As shown in Fig. S1(a), for $n < 0.1$ e/f.u., the in-plane lattice constants a and b of the $P4mm$ phase increase with increasing n whereas the out-of-plane lattice constant c decreases. As a result, the lattice of $P4mm$ $BaTiO_3$ evolves into the cubic phase when $n > 0.1$ e/f.u. This picture is confirmed when plotting the relative energy between the $P4mm$ and $Pm\bar{3}m$ phases of $BaTiO_3$, shown in Fig. S1(b). In the dark ($n = 0$ e/f.u.), $P4mm$ $BaTiO_3$ has a lower energy, but the energy difference decreases with increasing n, and both structures become degenerate for $n > 0.1$ e/f.u., when $P4mm$ $BaTiO_3$ transforms into the $Pm\bar{3}m$ phase.

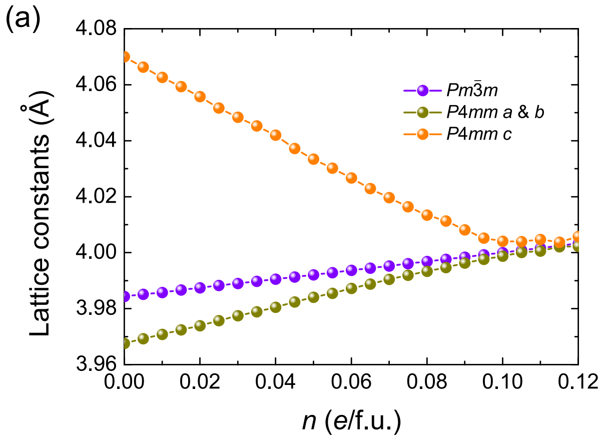

Figure S1: **Relative stability of $P4mm$ and $Pm\bar{3}m$ $BaTiO_3$ under illumination.** (a) Lattice constants of $P4mm$ and $Pm\bar{3}m$ $BaTiO_3$ under illumination. (b) Relative energy difference between the two phases with the energy of $Pm\bar{3}m$ $BaTiO_3$ set to be zero.

Topological phonons in room temperature $BaTiO_3$

Fig. S2(a) shows the calculated phonon dispersion of the room-temperature tetragonal phase of $BaTiO_3$ with and without LO-TO splitting. In the absence of LO-TO splitting, Fig. S2(b) shows that the Brillouin zone hosts two nodal rings located on the $q_x = 0$ and $q_y = 0$ planes (orange circles), four nodal lines located on the $q_x = \pi$ and $q_y = \pi$ planes (red lines), one nodal line along the Γ-Z high-symmetry line (black line), and two triple Weyl nodes along the Γ-Z high-symmetry line (orange dots). The two triple Weyl nodes are formed by the intersections of the two nodal rings on the $q_x = 0$ and $q_y = 0$ planes and the nodal line along the Γ-Z direction.

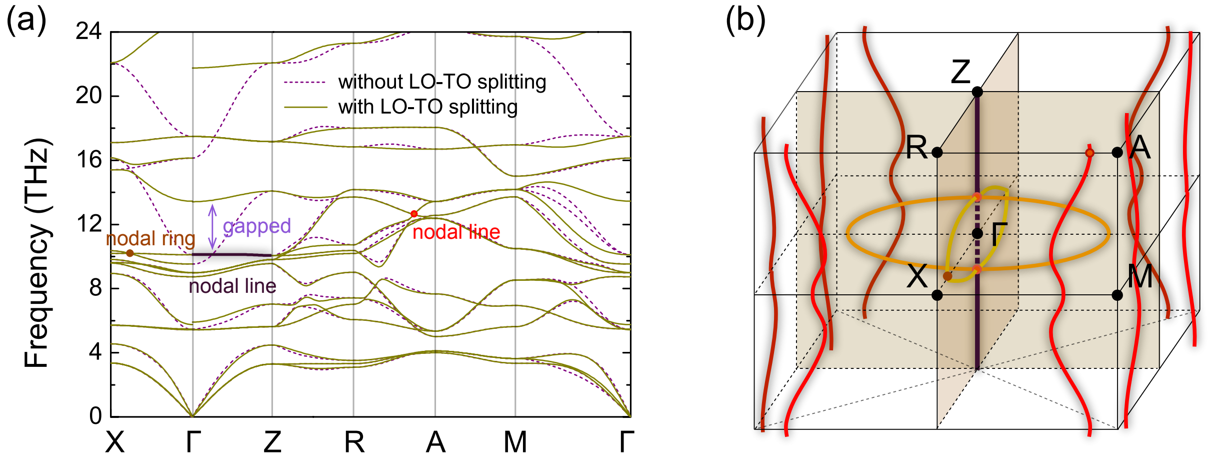

Figure S2: **Topological phonons in room temperature $BaTiO_3$.** (a) Phonon dispersion of tetragonal $BaTiO_3$ at 300 K with and without LO-TO splitting. (b) Bulk Brillouin zone (in the absence of LO-TO splitting) with two nodal rings on the $q_x = 0$ and $q_y = 0$ planes (orange circles), four nodal lines on the $q_x = \pi$ and $q_y = \pi$ planes (red lines), and one nodal line along the Γ-Z high-symmetry line (black line). All the band crossings are formed by the 10th and 11th branches.

When including LO-TO splitting, the non-analytical term separates the high-energy phonon branches near the Γ point [as indicated by the purple arrow in Fig. S2(a)] and as a result band crossings become more difficult and the triple Weyl points along the Γ-Z high-symmetry line no longer exist. By contrast, the $q_x = \pi$ and $q_y = \pi$ planes are far from the Γ point, so the nodal lines they host remain largely unchanged in the presence of LO-TO splitting.

Topological phonons in $Amm2$ $BaTiO_3$

The $Amm2$ phase of $BaTiO_3$ is never properly thermodynamically stable under photoexcitation. However, its energy is very close to those of the $R3m$ and $P4mm$ phases for carrier densities in the range $0.035 < n < 0.040$ e/f.u. (*39*) over which photoexcitation also dynamically stabilizes it. Fig. S3(a) shows the phonon dispersion of $Amm2$ $BaTiO_3$ at 0.04 e/f.u. The band crossings from the 13th and 14th bands along the R-Z and Z-T high-symmetry lines belong to a nodal ring that sits on the $q_z = \pi$ plane, while the crossing along the Z-Γ high-symmetry line is part of a nodal ring on the $q_x = -q_y$ plane, as shown in Fig. S3(b). In addition to the nodal rings, there are two pairs of Weyl points on the $q_x = q_y$ plane. These two pairs of Weyl nodes will annihilate with each other when $n < 0.035$ e/f.u. We note that the Brillouin zone of the $Amm2$ phase resembles that of the cubic phase as the $Amm2$ phase can be interpreted as a small deformation along the (011) direction of the cubic phase.

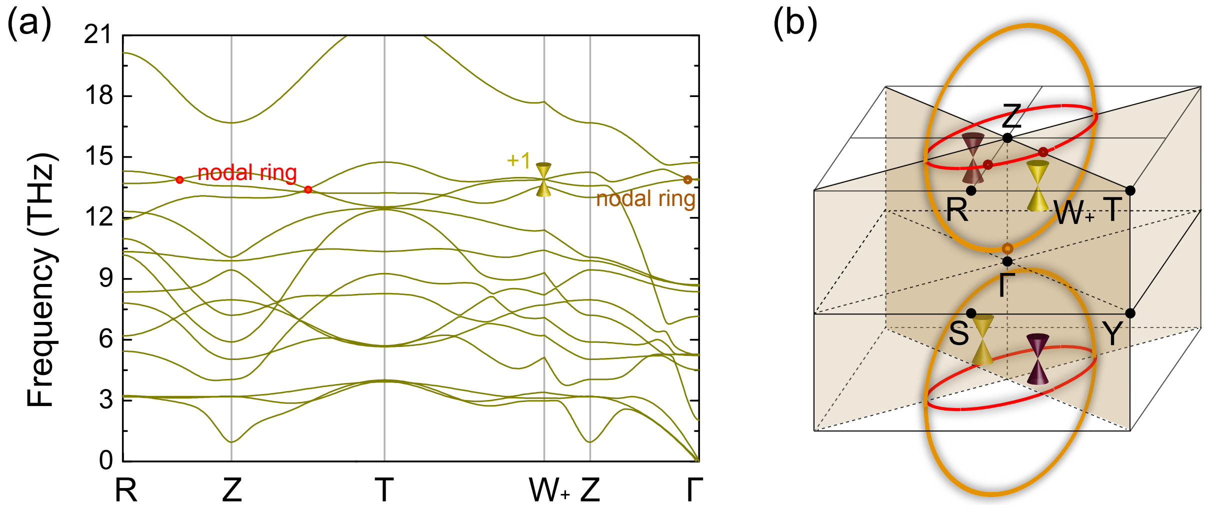

Figure S3: **Topological phonons in $Amm2$ $BaTiO_3$.** (a) Phonon dispersion of $Amm2$ $BaTiO_3$ at 0.04 e/f.u. (b) Bulk Brillouin zone with two nodal rings on the $q_x = -q_y$ plane and the $q_z = \pi$ plane respectively, and two pairs of Weyl points on the $q_x = q_y$ plane.

Topological phonons in $R3m$ $BaTiO_3$

The $R3m$ phase of $BaTiO_3$ is thermodynamically stable for photoexcited carrier densities $n < 0.035\,e$/f.u. (*39*). Fig. S4(a) shows the phonon dispersion of $R3m$ $BaTiO_3$ at 0.025 e/f.u. The band crossing of the 10th and 11th bands at the T point belongs to a nodal line [yellow straight line in Fig. S4(b)], protected by the C_3 rotation axis along the (111) direction (corresponding to the z axis in the Cartesian coordinate system). This yellow nodal line forms three nodal rings together with another three red loops located on the $q_y = 0$ mirror plane and the other two associated mirror planes rotated by $\pm 2\pi/3$ due to C_{3z} [Fig. S4(b)]. Their intersections form one pair of triple Weyl points. We note that the Brillouin zone of the $R3m$ phase in Fig. S4(b) resembles that of the cubic phase as the $R3m$ phase can be viewed as a small elongation of the cubic phase along the (111) direction, as shown in Fig. S4(c). There are 24 Weyl points formed by band crossings of the 10th and 11th bands, which can be cataloged into four groups according to their locations on four q_z planes. Each group is located either on the $q_z = \pm 0.111\pi$ planes (open circles) or on the $q_z = \pm 0.165\pi$ planes (solid dots) [Fig. S4(d)].

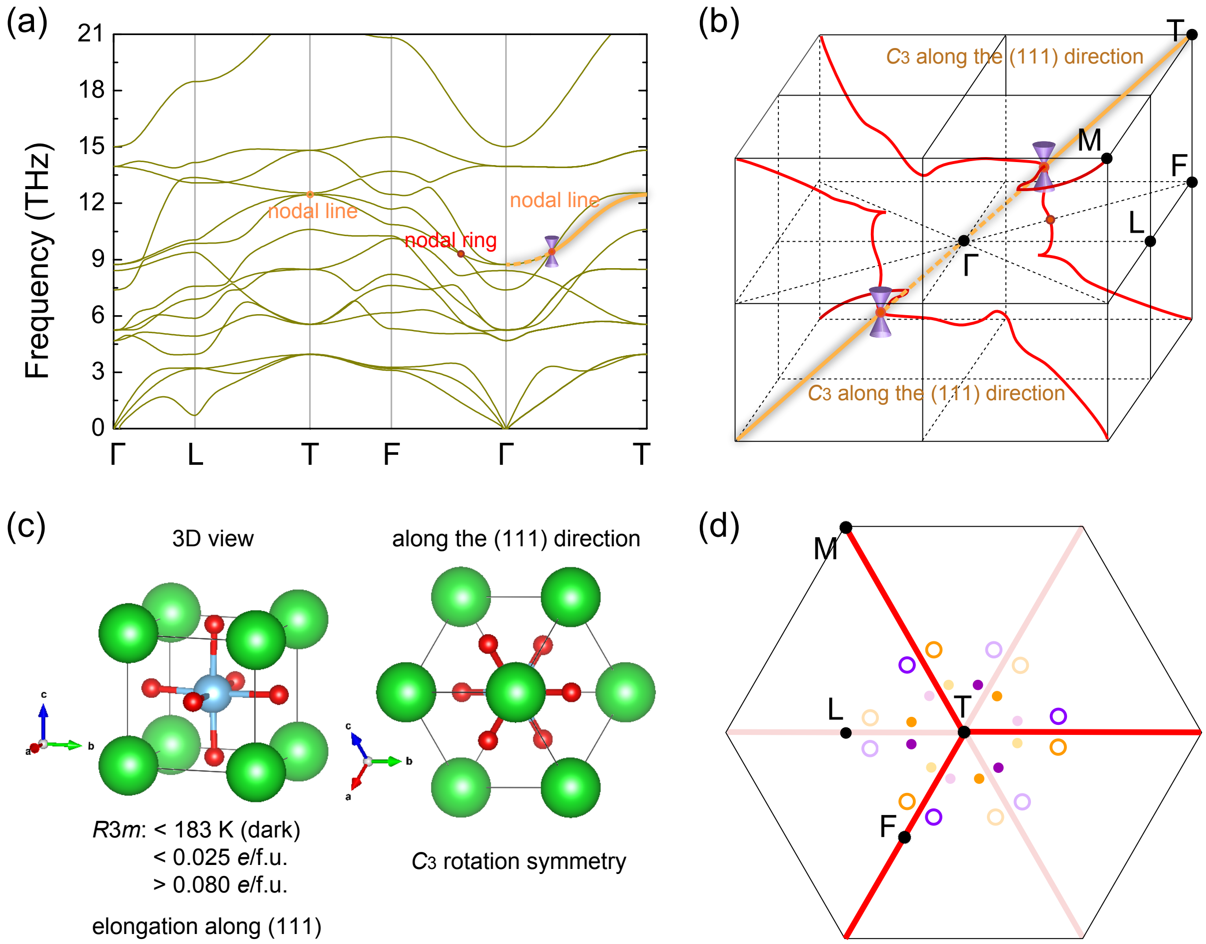

Figure S4: **Topological phonons in** $R3m$ **BaTiO$_3$.** (a) Phonon dispersion of $R3m$ BaTiO$_3$ at 0.025 e/f.u. (b) Bulk Brillouin zone with three nodal rings on three mirror planes crossing the C_{3z} axis (along the Γ-T high-symmetry line), and one pair of triple Weyl points at their intersection points. (c) Crystal structure of $R3m$ BaTiO$_3$. (d) View of the bulk Brillouin zone along the (111) direction with three red (light red) nodal rings on three mirror planes for $q_z > 0$ ($q_z < 0$) crossing the C_{3z} axis, related with each other by a rotation of $\pm 2\pi/3$ around the C_{3z} axis. There are also 12 pairs of Weyl points located on the $q_z = \pm 0.111\pi$ plane (open circles) and the $q_z = \pm 0.165\pi$ plane (solid dots), respectively.

Topological phonons in $P4mm$ BaTiO$_3$ with fixed in-plane lattice constants

A commonly used tuning parameter to study perovskite oxides is epitaxial strain, experimentally realized by growing the sample on appropriate substrates. As shown in Fig. S5(a), fixing the in-plane lattice constants makes the $P4mm$ phase thermodynamically more stable than the $Pm\bar{3}m$ phase in a much larger photoexcited carrier density range than in the corresponding fully relaxed case. Fig. S5(b) shows the change in lattice constant c of the $P4mm$ phase of BaTiO$_3$ with fixed in-plane strain as a function of n. Therefore, strain engineering provides an additional degree of freedom to tune the topological features of the phonon spectrum of BaTiO$_3$.

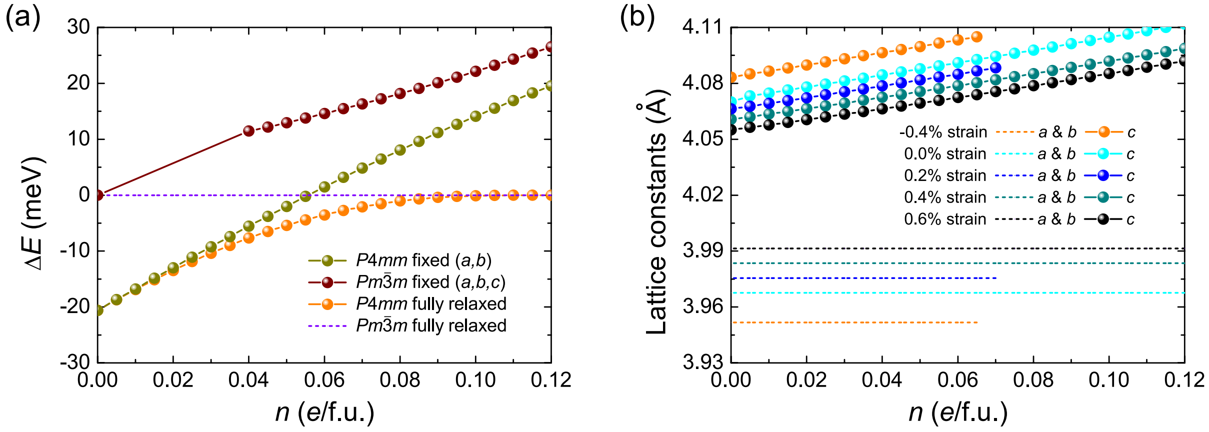

Figure S5: **Relative stability of $P4mm$ and $Pm\bar{3}m$ BaTiO$_3$ with fixed in-plane lattice constants from the dark.** (a) Relative energy difference between the $P4mm$ and the $Pm\bar{3}m$ phases with and without fixed in-plane lattice constants. The energy of fully relaxed $Pm\bar{3}m$ BaTiO$_3$ is set to be zero. (b) Lattice constants of $P4mm$ BaTiO$_3$ with fixed in-plane lattice constants under illumination.

Here we only show one example: we fix the in-plane lattice constants to those of the $P4mm$ phase of BaTiO$_3$ in the dark, which is equivalent to applying negative strain in the illuminated sample. Tetragonal BaTiO$_3$ with fixed in-plane lattice constants from the dark is dynamically stable between $n = 0.025 - 0.055$ e/f.u. As shown in Fig. S6(a)-(b), the 11th and 12th bands form four nodal rings on the $q_{x,y} = \pi$ planes, one nodal line along the Γ-Z high-symmetry line, and four pairs of Weyl points on the $q_z = 0$ plane. Different band compositions make the four

nodal rings lying on the $q_{x,y} = \pi$ planes protected by M_x and M_y symmetry. The surface arcs are shown in Fig. S6(c).

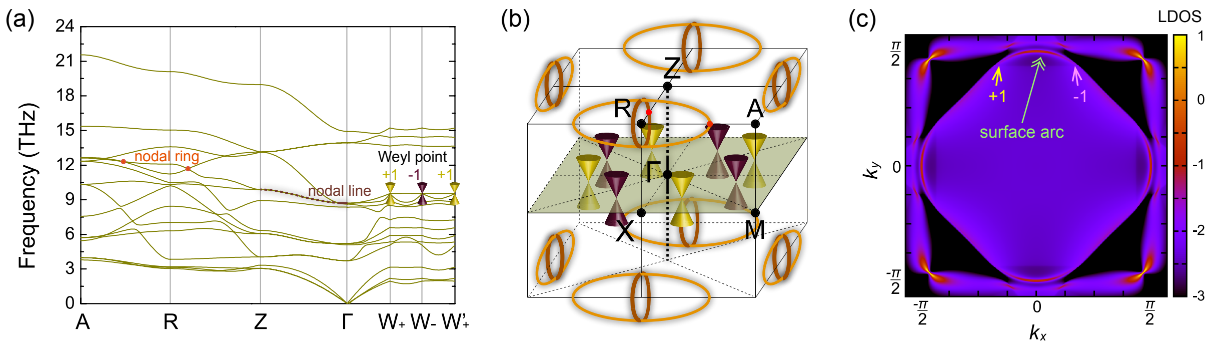

Figure S6: **Topological phonons in $P4mm$ $BaTiO_3$ with fixed in-plane lattice constants.** (a) Phonon dispersion of $P4mm$ $BaTiO_3$ at 0.05 e/f.u. with fixed in-plane lattice constants from the dark. (b) Bulk Brillouin zone with four nodal rings on the $q_{x,y} = \pi$ planes, one nodal line along the Γ-Z high-symmetry line, and four pairs of Weyl points on the $q_z = 0$ plane. (c) Phonon surface arcs for a phonon frequency of 9.54 THz at 0.05 e/f.u.

Topological phonons in $P4mm$ $BaTiO_3$ with fixed lattice constants

The in-plane lattice constants a and b of $P4mm$ $BaTiO_3$ increase upon photoexcitation, whereas the out-of-plane c decreases. Illuminated $BaTiO_3$ with fixed lattice constants (a, b, c) corresponds to a system with in-plane negative strain and out-of-plane positive strain. Such fixed lattice constants can be realized with a stoa-like structure consisting of stylobate (substrate) and column (matrix) with appropriately matched lattice constants.

When fixing the lattice constants of $P4mm$ $BaTiO_3$ to those of the dark, upon photoexcitation the lattice is dynamically stable between $n = 0.025 - 0.060$ e/f.u., and the Weyl points formed by the 10th and 11th bands no longer exist. Therefore we need to search among other band crossing points formed by other phonon branches. The most clearly visible Weyl points are formed by the 13th and 14th bands at 0.060 e/f.u. As shown in Fig. S7(a)-(b), there are four nodal rings on the $q_x = \pm q_y$ planes, one nodal line along the Γ-Z high-symmetry line, and four pairs of Weyl points on the $q_z = 0$ plane. The surface arcs are clearly visible [Fig. S7(c)].

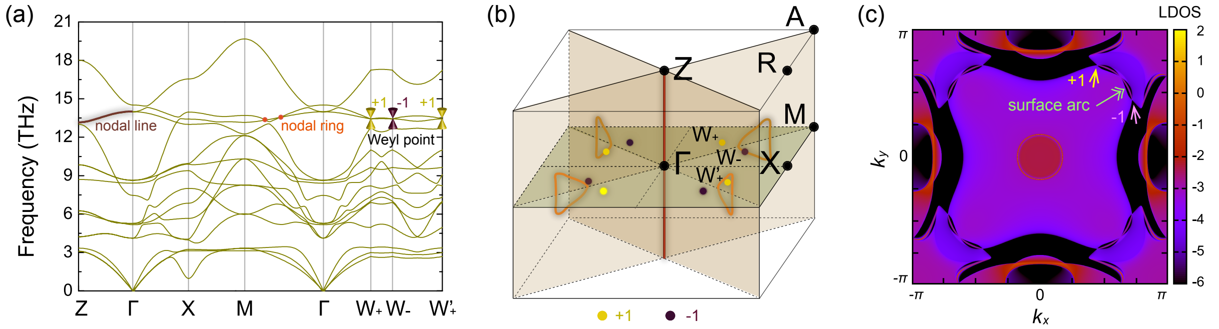

Figure S7: **Topological phonons in $P4mm$ $BaTiO_3$ with fixed lattice constants.** (a) Phonon dispersion of $P4mm$ $BaTiO_3$ at 0.06 e/f.u. with lattice constants fixed from the dark and internal coordinates fully relaxed. (b) Bulk Brillouin zone with four nodal rings on the $q_x = \pm q_y$ planes, one nodal line along the Γ-Z high-symmetry line, and four pairs of Weyl points on the $q_z = 0$ plane. (c) Phonon surface arcs for a phonon frequency of 13.43 THz at 0.06 e/f.u.

$PbTiO_3$ under illumination

$PbTiO_3$ has a tetragonal $P4mm$ structure for $T < 600\,\mathrm{K}$, and a cubic structure above that temperature. It has been reported that the $P4mm$ phase has the lowest energy among other phases in a wide photoexcited density range $n < 0.125$ e/f.u. (*39*). Therefore, we only focus on the structural and lattice dynamical properties of the $P4mm$ phase under illumination. As shown in Fig. S8(a), increasing photoexcited carrier density n leads to an increase of the in-plane lattice constants a and b of the $P4mm$ phase and to a decrease of the out-of-plane lattice constant c, similarly to what happens in $BaTiO_3$. The tetragonal phase is dynamically stable in the dark [dark yellow line in Fig. S8(b)], and photoexcitation leads to a lattice instability when $n > 0.05$ e/f.u. [purple dashed line in Fig. S8(b)].

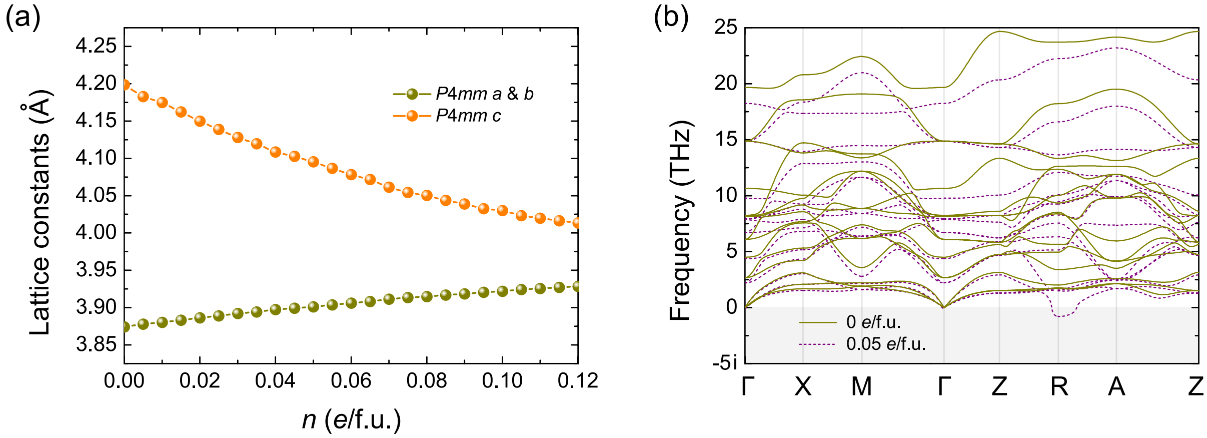

Figure S8: **$PbTiO_3$ under illumination.** (a) Lattice constants of $P4mm$ $PbTiO_3$ under illumination. (b) Phonon dispersion of tetragonal $PbTiO_3$ at $n = 0$ and 0.05 e/f.u.

Phonon spectra of cubic $BaTiO_3$ from ΔSCF and constrained DFT

The ΔSCF method used in the main text is computationally less demanding compared to other approaches like constrained DFT (*75*) and excited-state force calculations (*76*). We find that the ΔSCF method gives qualitatively similar phonon spectra compared to the constrained density functional theory calculations reported in Ref. (*39*), as shown in Fig. S9. However, there are some quantitative differences: comparable phonon spectra in the two methods are obtained with different photoexcited carrier densities. For example, the ΔSCF phonon dispersion at 0.095 e/f.u. provides the best match with the constrained DFT phonon dispersion at 0.15 e/f.u. [red curves in Fig. S9(a) and (b)]. This difference arises because the constrained DFT approach implements the quasi-Fermi level in a self-consistent manner, allowing for a relaxation of the band structure, which usually implies that higher concentrations are needed to observe the same phenomena. Despite these quantitative differences, both methods provide consistent features in the phonon dispersion, and therefore the results presented in the main text should be robust.

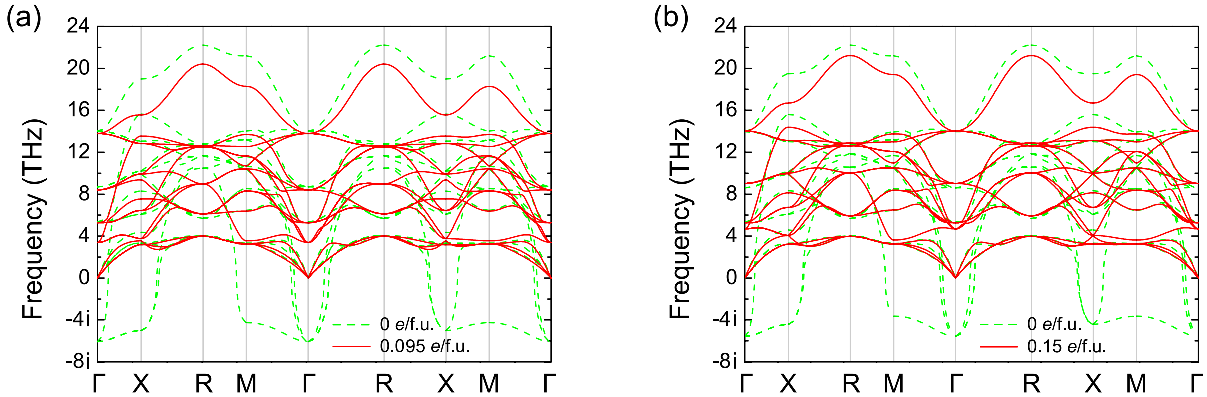

Figure S9: **Phonon spectra of cubic $BaTiO_3$ from ΔSCF and constrained DFT.** (a) Phonon dispersion of cubic $BaTiO_3$ at $n = 0$ and 0.095 e/f.u. using the ΔSCF method. (b) Phonon dispersion of cubic $BaTiO_3$ at $n = 0$ and 0.15 e/f.u. using constrained DFT taken from Ref. (*39*).
